# Bioinformatics method combined with logistic regression analysis reveal potentially important miRNAs in ischemic stroke

**DOI:** 10.1042/BSR20201154

**Published:** 2020-08-17

**Authors:** Zhiqiang Wei, Xingdi Qi, Yan Chen, Xiaoshuang Xia, Boyu Zheng, Xugang Sun, Guangming Zhang, Ling Wang, Qi Zhang, Chen Xu, Shihe Jiang, Xiulian Li, Bingxin Xie, Xiaohui Liao, Ai Zhu

**Affiliations:** 1 Department of Neurology, The Second Hospital of Tianjin Medical University, Tianjin 300211, P.R. China; 2 Public Administration, The Second Hospital of Tianjin Medical University, Tianjin 300211, P.R. China; 3 Department of Geriatric, The Second Hospital of Tianjin Medical University, Tianjin 300211, P.R. China

**Keywords:** ischemic stroke, machine learning algorithms, miRNAs

## Abstract

**Purpose:** The present study aimed to investigate the comprehensive differential expression profile of microRNAs (miRNAs) by screening for miRNA expression in ischemic stroke and normal samples.

**Methods:** Differentially expressed miRNA (DEM) analysis was conducted using *limma* R Bioconductor package. Target genes of DEMs were identified from TargetScanHuman and miRTarBase databases. Functional enrichment analysis of the target genes was performed using *clusterProfiler* R Bioconductor package. The miRNA-based ischemic stroke diagnostic signature was constructed via logistic regression analysis.

**Results:** Compared with the normal cohort, a total of 14 DEMs, including 5 up-regulated miRNAs and 9 down-regulated miRNAs, were identified in ischemic stroke patients. These DEMs have 1600 regulatory targets. Using a logistic regression model, the top five miRNAs were screened for constructing an miRNA-based ischemic stroke diagnostic signature. Using the miRNA–mRNA interaction pairs, two target genes (specificity protein 1 (*SP1*) and Argonaute 1 (*AGO1*)) were speculated to be the primary genes of ischemic stroke.

**Discussion and conclusion:** Here, several potential miRNAs biomarkers were identified and an miRNA-based diagnostic signature for ischemic stroke was established, which can be a valuable reference for future clinical researches.

## Introduction

Stroke is an acute predominant cerebrovascular disease, which causes brain tissue damage owing to a sudden rupture of blood vessels in the brain or the inability of blood to flow into the brain because of vascular occlusion [1]. In 2010, the incidence of stroke was approximately 16.9 million, which added to a pool of 33 million stroke survivors worldwide. Currently, stroke is considered to be the second leading cause of death after ischemic heart disease [2]. Typical symptoms of stroke include sudden unilateral weakness, numbness, visual loss, diplopia, altered speech, ataxia, and non-orthostatic vertigo [3,4]. Stroke can be categorized into ischemic and hemorrhagic stroke. The incidence of ischemic stroke is higher than that of hemorrhagic stroke, accounting for 60–70% of the total number of strokes [5,6]. Cardioembolic stroke accounts for approximately 25–30% of ischemic stroke cases, and 25% of such cases pass all diagnostic tests and have no known causes. Because of the acute onset, treatment difficulty, and the lack of detection methods, such cases pose a great challenge to clinicians.

MicroRNAs (miRNAs) are a class of small endogenous RNAs that regulate gene expression post-transcriptionally and play a role in gene silencing and translation inhibition by binding to target genes. The miRNAs are a highly conserved class of tissue-specific genes that have been found in all eukaryotic cells preserved across species since their discovery in 1993 [7,8]. In general, they are short RNA molecules measuring 19–25 nucleotides in size. A single miRNA can target hundreds of mRNAs and influence the expression of many genes often involved in a functional interacting pathway [9]. Appropriate maintenance of miRNA expression is required for a balanced physiological environment because these small molecules influence almost every genetic pathway from cell cycle checkpoint and cell proliferation to apoptosis, with a wide range of target genes [10]. In recent years, miRNA regulation has been extensively studied for their role in biological processes (BPs) as well as in the development and progression of various human diseases including ischemic stroke [11,12].

In mammals, the brain exhibits a high level and activity of several miRNAs that show region-specific expression [13,14]. Studies conducted using conditional knock-out Dicer (RNA enzymes are critical for miRNAs biogenesis) have demonstrated the indispensable functional significance of miRNAs in controlling processes that include cellular differentiation, proliferation, synaptic morphogenesis, and vascular formation [15–17], indicating an overlap between ischemic stroke and miRNAs. It has been suggested that stroke alters the expression levels of many miRNAs in human blood and brain [18,19].

Despite decades of research, treatment for ischemic stroke is limited to thrombolytic therapy and symptom management. Moreover, a considerable number of patients remain asymptomatic and cannot be detected at onset. To this end, a more comprehensive approach to predict potential ischemic stroke patients based on the differential expression of specific miRNAs is required. To address these issues, we performed bioinformatics analysis combined with machine learning algorithms to identify potential candidate diagnostic miRNAs. The present study will help us screen for ischemic stroke-associated miRNA biomarkers and can be tremendously useful for ischemic stroke patients.

## Materials and methods

### Study materials

The materials used in the present study were obtained from the Gene Expression Omnibus (GEO, https://ncbi.nlm.nih.gov/geo) with the accession number of GSE55937, which included 24 blood samples from healthy individuals and 24 blood samples from ischemic stroke. The miRNA expression profiles of the abovementioned samples were detected based on Affymetrix Multispecies miRNA-3 Array chip platform.

### Differential expression analysis

The miRNA expression profiles were normalized using robust multi-array (RMA) method via the *affy* R Bioconductor package and standardized by logarithmic transformation. Differentially expressed miRNAs (DEMs) were screened using the *limma* R Bioconductor package by employing the criteria of absolute log-transformed fold change (|log2FC|) > 0.5 and *P*-value ≤0.05.

### Construction of miRNA–mRNA regulatory network

The target genes of DEMs were searched from TargetScan (Release 7.2: March 2018 www.targetscan.org) and miRTarBase (Release 7.0: 15 September 2017; mirtarbase.mbc.nctu.edu.tw). Target genes that were common between the two databases were used for constructing the miRNA–mRNA regulatory network. Cytoscape software was applied for visualizing the regulatory network.

### Functional enrichment analysis

Functional enrichment analysis of the target genes of DEMs was conducted using clusterProfiler Bioconductor package. Ultimately, Gene Ontology (GO, including BP, Molecular Function, and Cellular Component) and KEGG pathways that satisfied Benjamini–Hochberg (BH)-adjusted *P*-value of <0.05 were retained.

### Construction of logistic regression model

Here, we proposed to test if DEMs could help distinguish stroke samples from normal ones. For this purpose, logistic regression analysis was performed by considering DEMs and sample groups as continuous predictor and categorical responsory, respectively, based on the *glm* basic R function. Each DEM with a *P*-value of <0.05 was retained for constructing the prediction model.

### Statistical analysis

Statistical analyses were performed using R software v3.5.2. *Affy* R Bioconductor package for the normalization of raw expression profiles. *Limma* R Bioconductor package was used for conducting differential expression analysis. A *P-*value of <0.05 was considered to be statistically significant in all of the abovementioned analyses.

## Results

### DEMs

**Figure 1:**
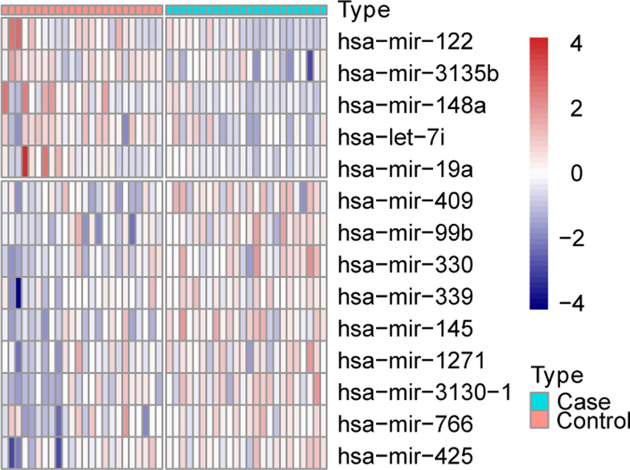
The overview of DEMs’ expression profile between ischemic stroke and normal samples Heat map display of the 14 DEMs in all samples. The horizontal axis is the sample and the vertical axis is miRNA. Red represents high expression and blue represents low expression.

Expression of all miRNAs contained in the miRNA chip after normalization is shown in Supplementary Figure S1A, which indicates that the normalization process successfully eliminated the batch effects. After calculation, we obtained a total of 14 DEMs in ischemic stroke samples in comparison with the normal samples, including 5 up-regulated miRNAs and 9 down-regulated miRNAs, as shown in Supplementary Figure S1B. Expressions of those 14 DEMs in normal and ischemic stroke groups were illustrated as a heatmap in Figure 1.

### Target genes of DEMs

**Figure 2:**
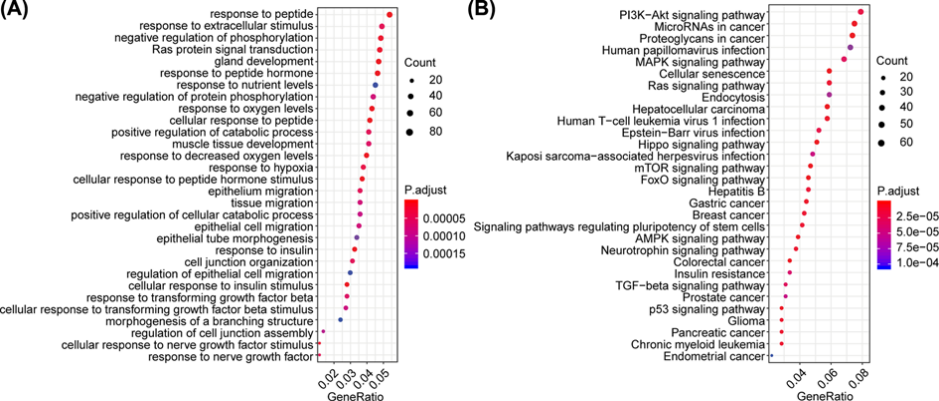
The GO and KEGG enrichment analysis of the 1600 target genes GO and KEGG enrichment results are shown in (**A**) and (**B**), respectively. The horizontal axis in the figure represents the ratio of genes enriched, and the vertical axis represents the name of each BP or pathway.

A total of 1600 target genes (Supplementary Table S1) were simultaneously predicted by TargetScanHuman and miRTarBase databases for 14 DEMs; functional enrichment analysis of these 1600 target genes led to the identification of a total of 499 and 87 significantly enriched GO terms, respectively. Figure 2A,B illustrates the top 30 most significant GO terms and KEGG pathways.

### DEMs effectively characterize ischemic stroke

**Figure 3:**
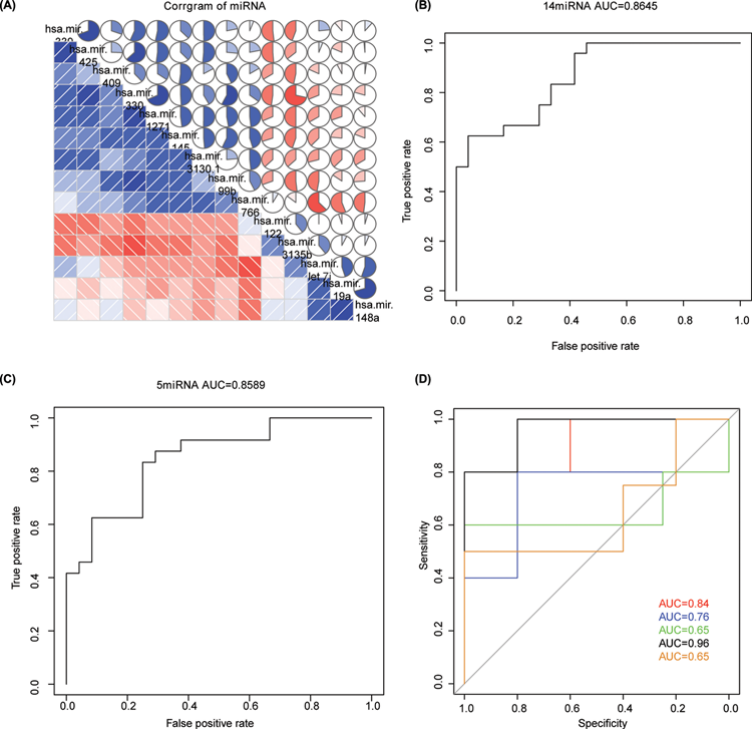
The collinear analysis and ROC curve of the DEMs (**A**) The collinear analysis of the 14 DEMs. The darker the blue or red color, and the larger the area of blue or red color in the Pie, the greater the collinearity between them. (**B**) The ROC curve of the model. The AUC value of the Logistic model was found to be 0.8645. (**C**) The top five miRNAs were used to reconstruct the logistic model with the AUC value of the logistic model reached 0.8589. (**D**) The ROC curve of the model basing five-fold cross-validation.

Correlation of expression of the 14 DEMs in ischemic stroke and normal samples is shown in Figure 3A, indicating that there was no particularly strong collinear relationship among them. Thus, all these 14 DEMs were used for the construction of logistic regression model. Receiver operating characteristic (ROC) analysis was used for evaluating the performance of the model. Consequently, the area under curve (AUC) value of the model was 0.8645, as shown in Figure 3B, which proved that the logistic model could robustly determine the sample type. More importantly, we found that the *P*-values of the five miRNAs, hsa_mir_122, hsa_mir_99b, hsa_mir_339, hsa_mir_145, and hsa_mir_3130_1, were less than 0.05, indicating that those five miRNAs had a greater contribution to the model than the remaining nine miRNAs. Hence, we reconstructed the logistic model using those five miRNAs, and it was found that the AUC value of the logistic model could reach 0.8589 (Figure 3C). Additionally, we conducted five-fold cross-validation basing on the dataset, and result illustrated high AUC value (Figure 3D). The abovementioned results proved that the model constructed based on these five miRNAs could effectively predict the sample type and should be more cost-effective for the diagnosis of ischemic stroke.

### Specificity protein 1 and Argonaute 1 are highly connected in miRNA–mRNA regulatory network

We constructed the miRNA–mRNA network for all 14 DEMs and the five DEMs that had a *P*-value of <0.05 in logistic regression analysis, as shown in Supplementary Figures S2A,B, respectively. Nodes in the network were colored according to their connectivity, i.e., number of their direct neighbors; furthermore, of the 1600 target genes, specificity protein 1 (*SP1*) and Argonaute 1 (*AGO1*) were the two genes that were regulated by at least two miRNAs in both regulatory networks. Therefore, they might be pivotal biomarkers in the development of ischemic stroke.

## Discussion

Ischemic stroke is a leading cause of death and disability, resulting in over 6 million deaths per year worldwide [20]. In addition to the high mortality rate, the timely monitoring of undiagnosed stroke patients is also a very critical issue. All these issues highlight the dire need for effective forecasting targets or biomarkers. Because miRNAs are implicated in a wide variety of diseases and have been shown to be essential for diverse proper physiological functions in the human brain, it is beneficial to develop a comprehensive specific expression profile of miRNAs in ischemic stroke patients for identifying potential miRNAs candidates as well as for targeting mRNA. Here, compared with the normal samples, a total of 14 DEMs in ischemic stroke patients were screened, including 5 up-regulated and 9 down-regulated miRNAs. Moreover, 1600 target genes have been further identified. We also studied the BPs related to these genes via GO and KEGG enrichment analysis. We found that these 1600 target genes were significantly enriched in ischemic stroke-related BPs, such as response to oxygen levels and response to decreased oxygen levels. The results of this biomarker selection study demonstrate not only the rationality of our method (many related BPs can be selected) but also the importance of two processes (response to oxygen levels and response to decreased oxygen levels) in ischemic stroke. The responses to oxygen levels and to decreased oxygen levels have been researched with regard to transport, oxygen homeostasis, translation, nitrogen fixation, and angiogenesis, which are involved in hypoxia, retinal neoplasms, tumor angiogenesis, retinoblastoma, and neoplasms [21,22]. A laboratory study conducted by Perez-Alvarez using an *in vivo* model revealed that mTORC1 (mammalian target of rapamycin complex-1), a protein complex downstream of PI3K-Akt pathway, was dysregulated after ischemic stroke and oxygen–glucose deprivation [23]. This evidence highlights the importance of understanding the relation between response to oxygen levels and ischemic stroke. Few other topics concerning response to oxygen levels and response to degraded oxygen levels in ischemic stroke may provide scope for more novel studies in the future.

We further reconstructed the logistic model and identified top five miRNAs (hsa_mir_122, hsa_mir_99b, hsa_mir_339, hsa_mir_145, and hsa_mir_3130_1). Previously, based on the screening of miRNA functional synergistic network, miR-145, miR-122, and miR-99b have been implicated to be associated with ischemic stroke by participating in the processes of post-ischemic neuronal damage and thrombosis, respectively, [24]. Based on our search, we found that the hsa_mir_339 and hsa_mir_3130_1 have not been well studied in ischemic stroke, which provide a very valuable starting point for future biomarker selection studies. The miR-339 and miR-3130 were mainly reported to be tumor suppressors in previous studies [25–27] owing to their biological roles in the suppression of cell proliferation. Additionally, Martinez et al. [28] also illustrated that miR-339 in the cerebellum and plasma of rats could be perturbed by *in vitro* stimulation with agents ethanol and caffeine; this indicates the potential of miR-339 as a novel biomarker for ischemic stroke. From the miRNA–mRNA regulatory network, two target genes (*SP1* and *AGO1*) are speculated to be the primary genes of ischemic stroke. AGO1 plays critical roles in RNA interference among the many regulators participating in miRNA formation. According to the findings reported by Shi et al., the expression of miR-103 is modulated by hypoxia-inducible factor 1α, which can target AGO1 to promote tumor vessel formation. Meanwhile, miR-103 can substantially affect angiogenesis and vascular density after ischemic stroke by targeting vascular endothelial growth factor (VEGF) [29]. SP1 is a member of a family of transcription factors that include SP2, SP3, and SP4; these factors are implicated in various essential BPs and have been established to play important roles in cell growth, differentiation, apoptosis, and carcinogenesis. SP1 reportedly interacts with zinc finger protein 179 (Znf179), which is a neuroprotective factor for the accumulation of reactive oxygen species (ROS). Znf179 autoregulation through Sp1-dependent mechanism plays an important role in neuroprotection, and NGF-induced Sp1 signaling may help attenuate more extensive (ROS-induced) damage following brain injury [30]. Both genes are related to ischemic stroke on some levels, and both those genes deserve to be investigationed further in detail.

In conclusion, in light of the fact that no gold standard treatment is currently available and that disease-specific prediction for ischemic stroke remains unreliable despite the presence of several standard criteria, we summarized the differential expression profile of miRNAs in ischemic stroke. The manner of expression of five miRNAs as well as of the two specific target genes (*SP1* and *AGO1*) may provide new insights into the discovery of therapeutic biomarkers. Because many pathological states are known to alter miRNA profiles and functions, understanding those changes and developing new target genes to rectify them might lead to the formulation of novel therapeutic strategies.

## Supplementary Material

Supplementary Figures S1-S2

Supplementary Table S1

## Abbreviations

AGO1: Argonaute 1
BP: biological process
DEM: differentially expressed miRNA
GO: Gene Ontology
KEGG: Kyoto Encyclopedia of Genes and Genomes
miRNA: microRNA
NGF: neuronal growth factor
ROS: reactive oxygen species
SP1: specificity protein 1
Znf179: zinc finger protein 179
