# Supplementary material for: Bioinformatics method combined with logistic regression analysis reveal potentially important miRNAs in ischemic stroke
Source: Biosci Rep. 2020 Aug 17;40(8):BSR20201154. doi: 10.1042/BSR20201154 (PMC7432999; doi:10.1042/BSR20201154)
Supplement: Supplementary Figures S1-S2 [file BSR-2020-1154_supp.pdf]

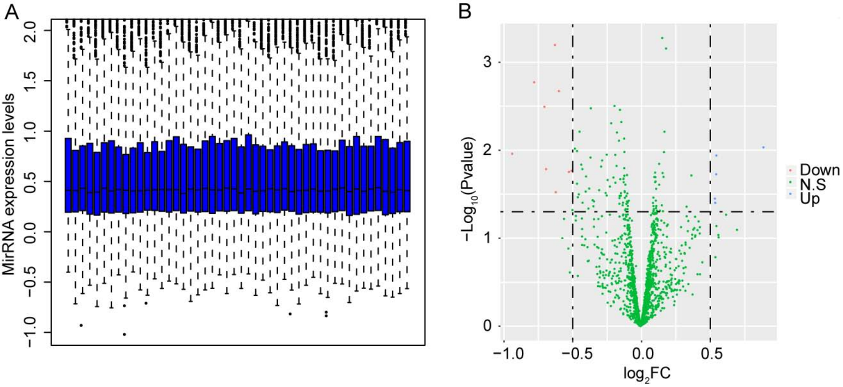
A
MirRNA expression levels
-1.0
-0.5
0.0
0.5
1.0
1.5
2.0
B
-Log10(Pvalue)
0
1
2
3
log2FC
-1.0
-0.5
0.0
0.5
Down
N.S
Up

Figure S1. Normalization of the raw miRNA expression profiles and differential expression miRNA analysis.(A) The distribution of miRNAs' expression values in each sample after the data normalization; the horizontal axis is the sample and the vertical axis is the miRNAs expression level. (B) Volcano plot of the differentially expressed miRNAs. The horizontal axis is the log2-based fold change (Log2FC) and the vertical axis is -log10 (P-value). The blue dots in the figure represent up-regulated miRNAs and the red dots represent down-regulated miRNAs. The green dots are non-differentially expressed miRNAs.

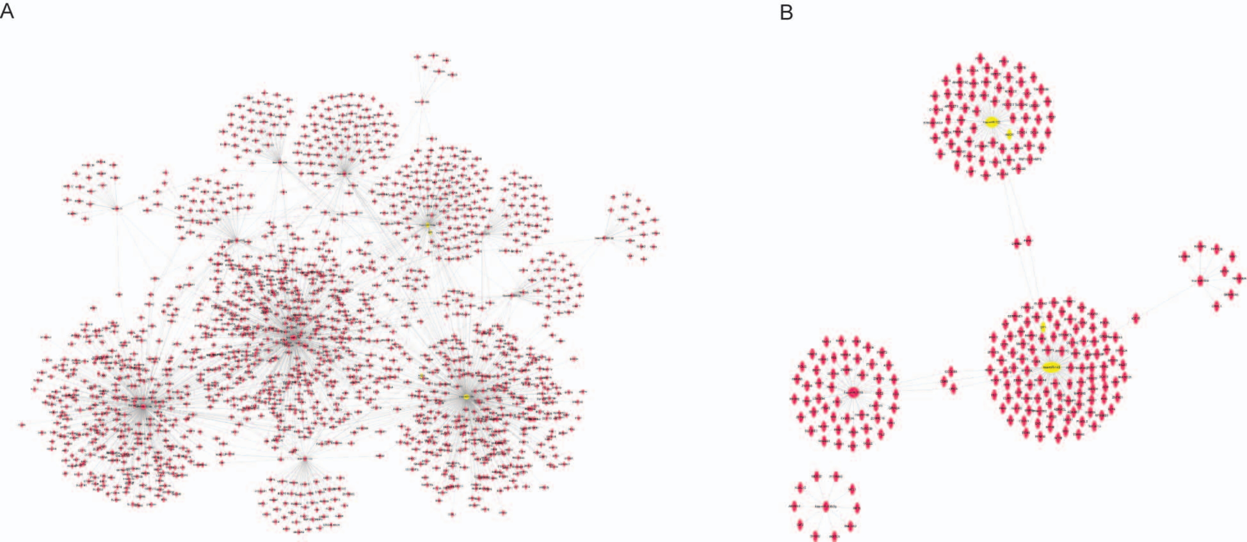
A
B

Figure S2. The prediction of miRNAs target genes. (A) The regulatory network between 14 miRNAs and target genes. (B) The regulatory network between 5 identified miRNAs and target genes. Each rectangle in the figure represents a node (miRNA or mRNA). SP1 and AGO1 as well as their regulatory miRNAs were highlighted in yellow.
